# Granulocyte colony-stimulating factor and autologous CD133-positive stem-cell therapy in liver cirrhosis (REALISTIC): an open-label, randomised, controlled phase 2 trial

**DOI:** 10.1016/S2468-1253(17)30326-6

**Published:** 2017-11-07

**Authors:** Philip Noel Newsome, Richard Fox, Andrew L King, Darren Barton, Nwe-Ni Than, Joanna Moore, Christopher Corbett, Sarah Townsend, James Thomas, Kathy Guo, Diana Hull, Heather A Beard, Jacqui Thompson, Anne Atkinson, Carol Bienek, Neil McGowan, Neil Guha, John Campbell, Dan Hollyman, Deborah Stocken, Christina Yap, Stuart John Forbes

**Affiliations:** a National Institute for Health Research Liver Biomedical Research Unit, University Hospitals Birmingham NHS Foundation Trust and the University of Birmingham, Birmingham, UK; b Centre for Liver Research, Institute of Immunology and Immunotherapy, University of Birmingham, Birmingham, UK; c Liver Unit, University Hospitals Birmingham NHS Foundation Trust, Birmingham, UK; d University of Birmingham, NIHR Liver BRU Clinical trials group, Cancer Research UK Clinical Trials Unit, Birmingham, UK; e Medical Research Council Centre for Regenerative Medicine, University of Edinburgh, Edinburgh, UK; f National Institute for Health Research Biomedical Research Unit in Gastrointestinal and Liver Diseases, Nottingham University Hospitals NHS Trust and the University of Nottingham, Nottingham, UK; g Cellular and Molecular Therapies, NHSBlood and Transplant, Birmingham, UK; h Scottish National Blood Transfusion Service, Edinburgh, UK; i Newcastle University, Newcastle Clinical Trial Unit, Institute of Health and Society, Newcastle, UK

## Abstract

**Background:**

Results of small-scale studies have suggested that stem-cell therapy is safe and effective in patients with liver cirrhosis, but no adequately powered randomised controlled trials have been done. We assessed the safety and efficacy of granulocyte colony-stimulating factor (G-CSF) and haemopoietic stem-cell infusions in patients with liver cirrhosis.

**Methods:**

This multicentre, open-label, randomised, controlled phase 2 trial was done in three UK hospitals and recruited patients with compensated liver cirrhosis and MELD scores of 11·0–15·5. Patients were randomly assigned (1:1:1) to receive standard care (control), treatment with subcutaneous G-CSF (lenograstim) 15 μg/kg for 5 days, or treatment with G-CSF for 5 days followed by leukapheresis and intravenous infusion of three doses of CD133-positive haemopoietic stem cells (0·2 × 10^6^ cells per kg per infusion). Randomisation was done by Cancer Research UK Clinical Trials Unit staff with a minimisation algorithm that stratified by trial site and cause of liver disease. The coprimary outcomes were improvement in severity of liver disease (change in MELD) at 3 months and the trend of change in MELD score over time. Analyses were done in the modified intention-to-treat population, which included all patients who received at least one day of treatment. Safety was assessed on the basis of the treatment received. This trial was registered at Current Controlled Trials on Nov 18, 2009; ISRCTN, number 91288089; and the European Clinical Trials Database, number 2009-010335-41.

**Findings:**

Between May 18, 2010, and Feb 26, 2015, 27 patients were randomly assigned to the standard care, 26 to the G-CSF group, and 28 to the G-CSF plus stem-cell infusion group. Median change in MELD from day 0 to 90 was −0·5 (IQR −1·5 to 1·1) in the standard care group, −0·5 (−1·7 to 0·5) in the G-CSF group, and −0·5 (−1·3 to 1·0) in the G-CSF plus stem-cell infusion group. We found no evidence of differences between the treatment groups and control group in the trends of MELD change over time (p=0·55 for the G-CSF group *vs* standard care and p=0·75 for the G-CSF plus stem-cell infusion group *vs* standard care). Serious adverse events were more frequent the in G-CSF and stem-cell infusion group (12 [43%] patients) than in the G-CSF (three [11%] patients) and standard care (three [12%] patients) groups. The most common serious adverse events were ascites (two patients in the G-CSF group and two patients in the G-CSF plus stem-cell infusion group, one of whom was admitted to hospital with ascites twice), sepsis (four patients in the G-CSF plus stem-cell infusion group), and encephalopathy (three patients in the G-CSF plus stem-cell infusion group, one of whom was admitted to hospital with encephalopathy twice). Three patients died, including one in the standard care group (variceal bleed) and two in the G-CSF and stem-cell infusion group (one myocardial infarction and one progressive liver disease).

**Interpretation:**

G-CSF with or without haemopoietic stem-cell infusion did not improve liver dysfunction or fibrosis and might be associated with increased frequency of adverse events compared with standard care.

**Funding:**

National Institute of Health Research, The Sir Jules Thorn Charitable Trust.

## Introduction

Chronic liver disease is a common cause of death globally, the incidence of which is rising due to a combination of alcohol consumption, obesity, and viral hepatitis.^1^, ^2^ Although the primary causes of injury, such as alcohol or viruses can be removed or treated, patients with cirrhosis often still have progression to liver decompensation leading to death.^3^ For such patients, the only proven treatment is liver transplantation, but access to this approach is limited globally by the shortage of donors, sequelae of long-term immunosuppression, and high lifelong medical costs.

Promising preclinical data have suggested that injections of bone-marrow-derived cells can reduce hepatic fibrosis and stimulate liver regeneration, thereby improving the synthetic function of the liver,^4^, ^5^, ^6^ although the mechanisms by which these effects are achieved have not been clearly elucidated.^7^ There have also been a series of proof-of-concept clinical studies, as reviewed by Moore and colleagues,^8^ in which infusions of bone-marrow-derived stem cells have potentially accelerated hepatic regeneration and improved liver dysfunction in the setting of liver fibrosis or cirrhosis.^9^, ^10^ However, these effects are not universally detected between studies and when seen are not always durable.^10^ Nevertheless, infusions of stem cells in patients with liver disease have been reported to be safe, except in studies in which cells were administered via the portal vein.^11^

**Research in context**

**Evidence before this study**

We (JM/SJF) searched MEDLINE and Embase in July, 2013, to find clinical studies involving patients with liver disease (any language) who had received autologous cellular therapy. To find relevant clinical studies we used the search terms “liver”, “cell”, “therapy”, “treatment”, “failure”, “decompensated”, “autologous”, “cell transplantation”, and “cell therapy”. Abstracts were assessed by two independent reviewers and the full text versions of studies that were relevant were analysed. Bibliographies of these papers and reviews were also studied, along with clinical trial websites (www.clinicaltrialresults.org and www.controlled-trials.com/ukctr) and abstract books from international liver conferences for the past 3 years. Studies chosen had to contain patients with chronic liver disease, who had received autologous stem cells (any route) along with outcome data covering safety and feasibility as a principal outcome. A range of secondary outcomes (prognostic liver scores, survival and changes in liver blood tests) were also looked at if relevant. Given the lack of randomised controlled studies, all trial designs were considered.

Most of the identified studies were small cohort, safety, and feasibility studies (median of ten treated patients) with fairly short follow-up (median 6 months). We found only six randomised controlled trials, but comment was not always made on how the patients were randomised; therefore, only one study was of good quality. There was heterogeneity across studies in outcome measures, causes of liver disease, dose, type, and route of cells given. Significant improvement in liver function tests was seen after treatment in 16 studies, suggesting that stem-cell administration might be associated with accelerated hepatic regeneration and improved liver dysfunction in liver fibrosis and cirrhosis. These effects were not universally observed across studies and when seen were not always durable. With the exception of studies in which cells were administered via the portal vein, infusions of stem cells in patients with liver disease were safe.

**Added value of this study**

To our knowledge, REALISTIC is the first multicentre, open-label, randomised, controlled phase 2 trial of either granulocyte colony-stimulating factor (G-CSF) alone or in conjunction with repeated infusions of purified autologous haemopoietic stem cells for patients with compensated chronic liver disease. The study was sufficiently powered to detect clinically important effects in liver function with the MELD score, which is the clinically recognised parameter for this purpose, and is predictive of future mortality. Our results showed that, in the setting of liver cirrhosis, no improvement in liver dysfunction or markers of liver fibrosis occurred after the administration of G-CSF or G-CSF plus stem-cell infusions. This study is of value to the field as it challenges the findings of other reports that show bone marrow cell therapy plus G-CSF to be effective for improving liver function in decompensated cirrhosis and acute-on-chronic liver failure. Our results also suggest that such therapies might even increase patient morbidity.

**Implications of all the available evidence**

The burgeoning clinical burden of chronic liver disease and absence of effective therapies has led to the consideration of innovative approaches, such as stem-cell therapies. Although this should be encouraged, it is important to ensure that decisions on the effectiveness of such new therapies are judged in the setting of robust randomised controlled trials that adhere to recognised regulatory standards. Our findings show that G-CSF with or without haemopoietic stem-cell infusions does not improve liver function or reduce liver fibrosis. Moreover, this approach might increase patients' risk of adverse events.

The size and nature of the trial designs of many of the previous studies make it impossible to draw meaningful conclusions on clinical outcomes, and thus the efficacy of bone-marrow-derived stem cells for liver cirrhosis has yet to be proven.^8^ In one adequately powered randomised controlled study, whole bone marrow mononuclear cells were infused into patients with acute inflammatory disease (alcoholic hepatitis), but had no effect.^12^ However, alcoholic hepatitis is a rarer and entirely different clinicopathological entity to that seen in patients with compensated liver cirrhosis, in which there is minimal inflammation and fibrosis is the major feature. Furthermore the mononuclear cells infused contained a mixed cell population.

Because liver cirrhosis is a chronic condition and a single dose of cells might have little effect, as suggested in a previous study^0^ we reasoned that multiple administrations of stem cells might have a greater antifibrotic effect, which was supported by our preclinical findings.^6^ We therefore designed a sufficiently powered, randomised controlled trial of granulocyte colony-stimulating factor (G-CSF) with or without repeated haemopoietic stem-cell (HSC) therapy in patients with compensated liver cirrhosis to detect clinically important effects on liver function and liver fibrosis and to assess safety.

## Methods

### Study design and participants

The REpeated AutoLogous Infusions of STem cells In Cirrhosis (REALISTIC) trial was a multicentre, open-label, randomised, controlled phase 2 trial done at three hospitals in Birmingham, Edinburgh, and Nottingham, UK. The National Research Ethics Service (NRES) Oxfordshire REC A committee (REC reference 09/H0604/64) and the Medicines and Healthcare Products Regulatory Agency (MHRA) approved all versions of the study protocol. Additionally, all recruitment sites obtained approval from their local hospital research and development departments. The University of Birmingham (Birmingham, UK) acted as the sponsor of the trial. A detailed version of the REALISTIC protocol has been published.^13^

The trial entry criteria were based on the presence of liver cirrhosis identified on biopsy or on clinical grounds as detailed in the appendix (p 3). Participants had to be aged 18–75 years with predominantly compensated cirrhosis (most causes were allowed except for autoimmune hepatitis) and a MELD score of 11·0–15·5.

Exclusion criteria were average alcohol ingestion greater than 21 units per week for men or greater than 14 units for women, any alcohol consumption within 6 months for patients with alcohol-related liver disease, ascites unless deemed by the investigator to be minimal and well controlled with no changes to diuretic therapy in the past 3 months, encephalopathy that was either current or required hospital admission for treatment in the past 3 months, portal hypertensive bleeding with an episode requiring treatment or hospital admission in the past 3 months, previous or current history of hepatocellular carcinoma, presence of dysplastic or indeterminate nodules, previous liver transplant (or being on a list for a transplant), and recent history of pulmonary infiltrates or pneumonia. All patients with hepatitis C virus (HCV) infection had to have received previous antiviral treatment before being considered for the trial, but none had cleared the virus after treatment and thus all had ongoing viral injury.

We recruited participants from among patients attending the liver clinics. All patients gave written informed consent, the study was done by site investigators, and data were gathered by specifically trained personnel.

### Randomisation

Centre-delegated staff telephoned randomisation officers at the Cancer Research UK Clinical Trials Unit (CRCTU; Birmingham, UK), who used a computer-generated, centrally administered procedure to randomly assign eligible patients (1:1:1) to one of three treatments: standard care alone, G-CSF, or G-CSF plus stem-cell infusion. Randomisation was based on a minimisation algorithm (prepared and validated by the CRCTU programming and statistical team) and patients were also stratified by trial site and cause of disease (alcohol-related liver disease, HCV, and other). Each patient was then allocated a unique patient trial number and scheduled for treatment and follow-up visits. The local site staff could not predetermine treatment allocation, the treatment allocation was open, and all clinicians and local site staff, as well as the patients, were aware of which treatment had been allocated (open-label therapy) following randomisation.

### Procedures

All patients received standard management for compensated cirrhosis, which could include disease-specific medications and treatments for the complications of cirrhosis. Patients assigned to the control group received standard of care management alone. Patients assigned to the G-CSF group received subcutaneous injections of G-CSF (lenograstim; Chugai Pharmaceuticals, London, UK) at 15 μg/kg bodyweight daily for 5 consecutive days. Patients assigned to receive G-CSF plus stem cells received subcutaneous injections of G-CSF 15 μg/kg bodyweight per day for 5 consecutive days and underwent leukapheresis and intravenous infusion of three doses of CD133-positive haemopoietic stem cells (HSCs; 0·2 × 10^6^ cells per kg) on days 5, 30, and 60 after randomisation. Isolation of CD133-positive HSCs from the harvested peripheral blood mononuclear cells was done under aseptic conditions within clean room facilities in accordance with Good Manufacturing Practice regulations (MHRA/HTA, UK). Cell sorting was done in a closed, sterile system to provide clinical grade enrichment (CliniMACS Plus, Miltenyi Biotec, Bergisch Gladbach, Germany).

Cells were tested with flow cytometry (CD45-positive, CD34-positive, and CD133-positive) on the day of isolation to establish positive and negative fractions. The gating strategy was set up to exclude debris, beads, and non-viable cells, before gating on CD45 and then CD133 expression such that total cells included were viable and positive for CD45 and CD133. This information was used to aliquot three doses 0·2 × 10^6^ cells per kg for each patient. Cells had to have greater than 50% viability, which is in line with clinical practice. Fresh CD133-positive HSCs were administered intravenously for immediate reinfusion and a further two aliquots were cryopreserved according to site standard protocols for later reinfusion at day 30 and day 60 post randomisation, thus requiring the administration of a minimum total number of cells of 0·6 × 10^6^ cells per kg. Cells for days 30 and 60 were supplied to the ward frozen and thawed before being administered; results for sterility testing from BacTalert bottles were provided at the same time, in line with standard clinical practice for the administration of HSCs to patients receiving stem cell transplants. If there were insufficient cells for three doses, cells were allocated preferentially to the first, then second, and then third dose. The procurement, processing, storage and distribution of the autologous CD133-positive HSCs was done in accordance with Tissue Quality and Safety Regulations by establishments holding Human Tissue Authority licences.

After randomisation, all patients returned for study visits at days 30, 60, 90, 180, and 360 (end of study). The coprimary outcomes used data captured from baseline to day 90. The schedule for the study visits and data collection is summarised in the appendix (p 5).

### Outcomes

The coprimary outcome measures were change in MELD score at 90 days from baseline and the trend of treatment activity established by incorporating MELD score measured at baseline and days 30, 60, and 90. The protocol was updated in March, 2015, to include the second coprimary outcome to make better use of data collected and detect differences before 90 days. MELD score is calculated from objective variables that are readily obtained: serum bilirubin, serum creatinine, and International Normalised Ratio (INR).^14^ Secondary outcome measures were liver stiffness (Fibroscan), enhanced liver fibrosis (ELF) test, Chronic Liver Disease Questionnaire (CLDQ) scores, individual components of liver function (bilirubin, albumin, INR, and creatinine values [creatinine was unplanned]), UK End-Stage Liver Disease (UKELD) score, circulating peripheral blood HSCs, long-term MELD and UKELD (to day 360), clinical events, and transplant-free survival.

Safety and adverse events were assessed with standard reporting forms by trained investigators. The reporting period for adverse events started from the date of patient consent and continued throughout the study until visit 7 (day 360). Serious adverse events were reported from date of consent until 30 days after the last possible stem-cell infusion (day 90) for all treatment groups, therefore ensuring that the reporting period stayed the same for all treatment groups. All serious adverse events and adverse events were assessed by the local investigators and recorded. We used the National Cancer Institute's common terminology criteria for adverse events (CTCAE version 4.02–2010) to grade each adverse event. The CRCTU kept detailed records of all adverse events reported (nature, onset, duration, severity, and outcome) and did an assessment with respect to seriousness, causality, and expectedness.

### Statistical analysis

The sample size calculation was based on the change in MELD score from baseline to 90 days after randomisation. With pooled standard deviation assumed to be 1·25 in this controlled setting, the trial aimed to detect a standardised effect size of at least 0·8 between treatment groups and the control group, which would equate to a 1 point reduction in MELD. To have error rates based on two-sided α of 0·05 (α=0·10 split equally between the two hypotheses) and 80% power, we needed to recruit 27 patients into each group.^13^ The trial was designed as a three-armed study with one control group and was powered to compare each treatment to control with respect to the first coprimary outcome, but was not powered to detect differences between the two treatment groups.

The hypothesis of the primary analysis was designed to assess activity and, as such, all analyses were done in the modified intention-to-treat (mITT) population. The mITT population included all participants who received at least one day of treatment (one day of G-CSF at 15 μg/kg bodyweight in the treatment groups, plus one infusion of at least 0·17 × 10^6^ cells per kg for the G-CSF plus stem-cell infusion group), with patients retained in their randomly assigned treatment groups, including those who violated the protocol or were ineligible. The per-protocol population was defined as any patients who received 5 days of G-CSF at an average daily dose of at least 12 μg/kg and any patients who received 5 days of G-CSF plus three infusions at a minimum of 0·17 × 10^6^ cells per kg each. All patients in the control group were included in the mITT and per-protocol populations. We defined the safety population on the basis of the actual treatment received.

We calculated the first coprimary outcome, change in MELD score from baseline to day 90, for each participant and we compared treatment groups with the control using the non-parametric two-sample Wilcoxon test. We fitted exploratory linear regression models, with transformed MELD scores where required, to enable adjustment of the estimates of the first coprimary outcome. For the second coprimary outcome, we explored the effect of treatment on the change in MELD score through linear mixed-effects models (details of the modelling approach used to find the most appropriate parsimonious model are shown in the appendix [p 7]), which incorporated measurements taken at baseline and at days 30, 60, and 90. We assigned random effects at the patient level. The first coprimary outcome was conditional on the availability of day 90 MELD measures, whereas the second coprimary outcome was not. Sensitivity analyses included adjustment of model-based analyses for known prognosticators, such as alcohol intake and cause of liver disease, minimisation factors, and differences at baseline.

We analysed UKELD in the same manner as the coprimary outcomes, and we assessed CLDQ responses through area-under-curve analyses with a set of varied assumptions applied to address censoring, and, as a sensitivity analysis, removing participants who had major adverse events. We also assessed long-term MELD and UKELD, measured to day 360, in the same way as the coprimary outcomes. We compared average change in liver parameters between treatment groups and the control group from baseline using a test appropriate to the data (*t* tests or Wilcoxon rank sum). We report other outcome measures descriptively. We used Stata version 14 for all analyses.

Data were collected and presented to the independent data management committee regularly throughout the recruitment, treatment, and follow-up periods of the study (appendix p 2). The committee advised the trial management team throughout the study in relation to trial conduct and specifically whether extra data monitoring was needed throughout the trial. The data monitoring committee operated in accordance with a trial-specific charter based on the template created by the Damocles Group. This trial was registered at Current Controlled Trials on Nov 18, 2009; ISRCTN, number 91288089; and the European Clinical Trials Database, number 2009-010335-41.

### Role of the funding source

The funders had no role in study design, data collection, data analysis, data interpretation, or writing of the report. The corresponding author had access to all data in the study and had final responsibility for the decision to submit for publication.

## Results

**Figure 1:**
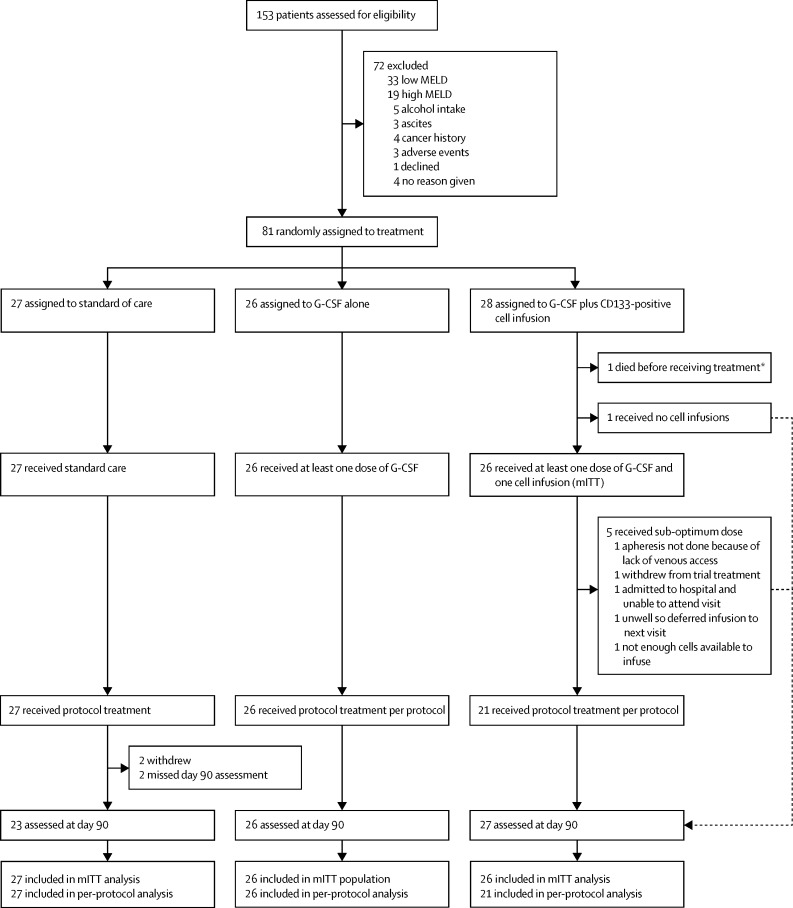
Trial profile We defined the mITT population as including any patient assigned to receive G-CSF who received a minimum of 1 day of G-CSF or any patient assigned to receive G-CSF plus stem-cell infusion who received at least one infusion of at least 0·17 × 10^6^ cells per kg. We defined the per-protocol population as including patients in the G-CSF group who attended all G-CSF visits and received an average daily dose of at least 12 μg/kg and patients in the G-CSF plus stem-cell infusion group who received 5 days of G-CSF and a cumulative cell infusion of at least 0·51 × 10^6^ cells per kg. All patients in the standard care group were included in the mITT and per-protocol populations. Because the second coprimary outcome allowed imputation of missing values, the mITT numbers are greater than the number of available day 90 values. mITT=modified intention to treat. G-CSF=granulocyte colony-stimulating factor. *Patient received no treatment so the event is attributed to the standard care group based on definition of the safety population.

Between May 18, 2010, and Feb 6, 2015, 153 patients with liver cirrhosis were screened, and 81 underwent randomisation (figure 1). We recruited 58 patients from the Birmingham centre, 19 from the Edinburgh centre, and four from the Nottingham centre. The 81 patients recruited were not equally allocated to the treatment groups, because the minimisation procedure allocated patients to ensure that groups were balanced with respect to the specified minimisation factors. The final patient's characteristics resulted in a balanced state and hence the allocation was made randomly to the G-CSF plus stem-cell infusion group. The mITT population included 27 patients in group 1, 26 patients in group 2, and 26 patients in group 3, whereas the per-protocol population included 27 patients in group 1, 26 patients in group 2, and 21 patients in group 3. In group 3, one patient received no G-CSF nor any cells (this patient later died after 2·3 months), one patient received G-CSF only (withdrew from study), and a further five patientsreceived G-CSF but did not receive sufficient cells to complete three full doses (appendix p 13).

At baseline, we noted differences between the treatment groups in terms of gender, urea, and creatinine (table 1). Patients had features of liver decompensation (ascites, encephalopathy, and prior variceal bleeding), although these were mild and responsive to standard medical treatment. The median MELD score was 13·1 (IQR 12·4–13·8) in the standard treatment group, 12·7 (12·0–13·1) in the G-CSF group, and 13·2 (12·1–13·9) in the G-CSF plus stem-cell infusion group. 22 patients had previous histological confirmation of liver cirrhosis.

**Table 1:** Baseline characteristics

		**Standard care (n=27)**	**G-CSF only (n=26)**	**G-CSF plus CD133-positive cell infusion (n=28)**
**Demographics**				
Age (years)		52·0 (47·0–60·0)	54·0 (49·0–61·0)	56·5 (47·5–62·5)
Gender				
	Male	13 (48%)	18 (69%)	22 (79%)
	Female	14 (52%)	8 (31%)	6 (21%)
BMI		27·9 (24·5–35·8)	27·4 (25·0–31·3)	30·6 (27·5–34·6)
**Cause of liver disease**				
Alcohol-related liver disease		12 (44%)	12 (46%)	14 (50%)
Hepatitis C		4 (15%)	3 (12%)	3 (11%)
Other causes		11 (41%)	11 (42%)	11 (39%)
	Non-alcoholic fatty liver disease	5 (19%)	3 (12%)	5 (18%)
	Primary biliary cholangitis	5 (19%)	7 (27%)	3 (11%)
	Cryptogenic	0 (0%)	1 (4%)	2 (7%)
	Mixed	1 (4%)	0	1 (4%)
**Liver disease severity**				
MELD		13·1 (12·4–13·8)	12·7 (12·0–13·1)	13·2 (12·1–13·9)
UKELD		51·5 (49·8–54·2)	51·1 (50·0–52·5)	52·0 (50·9–53·5)
Child-Pugh score		7·0 (7·0–8·0)	7·0 (6·0–8·0)	7·0 (6·0–8·0)
Child-Pugh (class)				
	A	6 (22%)	7 (27%)	11 (39%)
	B	20 (74%)	19 (73%)	16 (57%)
	C	1 (4%)	0	0
	Unknown	0	0	1 (4%)
**Liver comorbidities**				
Ascites		13 (48%)	10 (39%)	14 (50%)
Variceal bleeding		7 (26%)	11 (42%)	11 (39%)
Encephalopathy		3 (11%)	3 (12%)	7 (25%)
**Medications**				
ACE inhibitor		1 (4%)	2 (8%)	3 (11%)
Angiotensin receptor antagonists		0	0	1 (4%)
Antiviral		0	0	0
Diuretic		8 (30%)	14 (54%)	14 (50%)
Lactulose		7 (26%)	7 (27%)	3 (11%)
Neomycin		0	1 (4%)	0
Rifaximin		2 (7%)	1 (4%)	0
Other		23 (85%)	23 (88%)	22 (79%)
**Full blood count**				
Haemoglobin (g/dL)		12·9 (11·8–13·8)	13·1 (11·6–14·3)	12·9 (12·1–14·3)
White blood cells (× 10^9^ per L)		4·2 (3·3–5·4)	4·3 (3·4–5·2)	4·3 (3·3–5·3)
Platelets (× 10^9^ per L)		77·0 (57·0–92·0)	90·5 (54·0–116·0)	78·5 (57·0–106·5)
**Biochemistry**				
Sodium (mmol/L)		140·0 (137·0–142·0)	140·0 (137·0–142·0)	139·0 (137·0–140·0)
Potassium (mmol/L)		3·9 (3·7–4·4)	4·0 (3·7–4·2)	4·1 (4·0–4·2)
Urea (mmol/L)		3·7 (2·7–4·2)	3·8 (2·9–4·8)	4·8 (3·7–5·3)
Creatinine (μmol/L)		62·0 (52·0–74·0)	63·0 (56·0–75·0)	71·0 (64·0–90·0)
**Liver function tests**				
Bilirubin (μmol/L)		38·0 (30·0–53·0)	44·0 (34·0–53·0)	41·5 (33·0–51·0)
Albumin (g/L)		33·0 (30·0–37·0)	36·0 (30·0–39·0)	35·5 (33·5–39·0)
Aspartate aminotransferase (U/L)		44·0 (35·0–62·0)	50·5 (37·0–82·0)	48·0 (37·0–62·0)
Alanine aminotransferase (U/L)		28·0 (20·0–39·0)	31·5 (21·0–54·0)	31·0 (21·5–45·0)
Alkaline phosphatase (U/L)		160·0 (108·0–255·0)	142·5 (118·0–282·0)	138·5 (97·5–244·0)
γ glutamyl transpeptidase (g/dL)		68·0 (49·0–110·0)	86·0 (57·0–198·0)	73·0 (41·0–188·5)
α fetoprotein (IU/L)		3·0 (2·0–6·0)	3·0 (2·0–5·0)	3·0 (2·0–5·0)
International Normalised Ratio		1·4 (1·2–1·4)	1·2 (1·2–1·4)	1·3 (1·2–1·4)
**Non-invasive hepatic biomarkers**				
Fibroscan (kPa)		32·5 (22·2–44·8)	34·3 (26·1–66·4)	28·9 (17·3–45·2)
ELF test				
	ELF score	12·0 (11·2–13·0)	11·9 (11·4–12·6)	12·1 (11·3–13·0)
	Hyaluronic acid	490·6 (265·2–879·9)	476·9 (253·8–722·2)	574·4 (366·8–807·5)
	Amino-terminal propeptide of type III procollagen	17·1 (12·0–22·8)	18·2 (13·0–25·7)	18·7 (13·2–26·4)
	Tissue inhibitor of metalloproteinases 1	329·3 (267·0–399·1)	372·2 (289·5–507·6)	322·9 (227·1–412·2)
**Quality of life score (CLDQ)**				
Abdominal		6·3 (4·7–7·0)	5·7 (4·0–6·7)	6·0 (4·7–7·0)
Fatigue		4·5 (2·8–6·0)	3·5 (2·6–5·2)	4·2 (3·6–5·2)
Systemic		5·4 (4·0–6·2)	5·4 (4·0–6·0)	5·2 (4·4–6·0)
Activity		6·0 (3·7–7·0)	4·7 (3·2–6·0)	5·8 (4·7–6·8)
Emotion		5·6 (4·5–6·8)	4·8 (3·7–5·8)	5·4 (4·5–6·1)
Worry		5·8 (3·9–6·9)	5·1 (3·3–6·6)	4·8 (3·6–6·4)
Overall		5·5 (3·4–6·3)	4·8 (3·5–5·5)	5·0 (4·2–6·1)

Data are n (%) or median (IQR). G-CSF=granulocyte colony-stimulating factor. ACE=angiotensin-converting enzyme. ELF=enhanced liver fibrosis. UKELD=UK End-Stage Liver Disease Score. CLDQ=Chronic Liver Disease Questionnaire.

In the mITT population, day 90 MELD scores were obtained from 23 of 27 patients in the standard care group, and all patients in each of the treatment groups (figure 1). Median change in MELD from day 0 to 90 was −0·5 (IQR −1·5 to 1·1) in the standard care group, −0·5 (−1·7 to 0·5) in the G-CSF group, and −0·5 (−1·3 to 1·0) in the G-CSF plus stem-cell infusion group. We found no evidence of a difference in change in MELD scores between days 0 and 90 in either of the treatment groups compared with the standard care group (p=0·72 for G-CSF *vs* standard care and p=0·90 for the G-CSF plus stem-cell infusion group *vs* standard care). Waterfall plots of day 90 change in MELD score and best change in MELD score are presented in the appendix (pp 15, 16).

**Figure 2:**
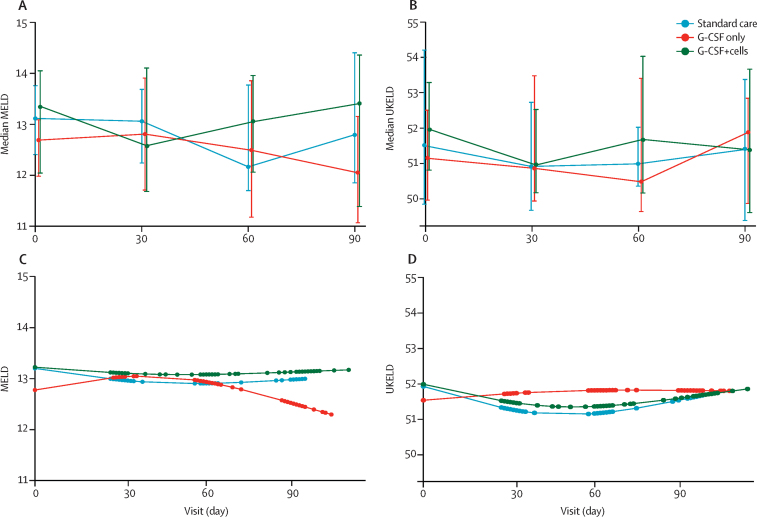
Change in MELD and UKELD scores Observed median change in (A) MELD and (B) UKELD scores at baseline and days 30, 60, and 90. Bars show IQR. Model fit based on predictions from a linear mixed-effects model incorporating restricted cubic splines for (C) MELD and (D) UKELD respectively. G-CSF=granulocyte colony-stimulating factor. UKELD=UK End-Stage Liver Disease Score.

Simple mixed-effects models showed no evidence of a trend in MELD over time (p=0·33 for all groups), nor of a difference between the treatment and control groups (p=0·55 for the G-CSF group *vs* the standard care group and p=0·75 for the G-CSF plus stem-cell infusion group *vs* the standard care group). The final, more flexible model for the second coprimary outcome included a change-point at day 30 and an interaction between treatment group and time to allow for different trends in each period (appendix p 7). Despite an improved fit, we found no evidence of a difference in trends in either model (figure 2). In the per-protocol population the median change in MELD score from day 0 to 90 was −0·5 (−1·5 to 1·1) in the standard care group, −0·5 (−1·7 to 0·5) in the G-CSF group, and −0·7 (−1·1 to 0·7) in the G-CSF plus stem-cell infusion group, with no evidence of differences between treatment and control groups (p=0·72 for G-CSF *vs* standard care and p=0·90 for G-CSF plus stem-cell infusion *vs* standard care). We found no evidence of any differences in analyses with the second coprimary outcome in the per-protocol population. Inferences were unchanged for both coprimary outcome measures when adjusted for participant characteristics, including cause of liver disease, alcohol consumption history, and the baseline imbalances between the groups (appendix pp 10–12).

We detected no evidence of any differences in UKELD scores between groups (figure 2), and no differences in either composite or individual markers of liver dysfunction across any of the groups (table 2). Moreover, we found no differences in markers of liver fibrosis (serum ELF or Fibroscan) or quality of life scores (CLDQ). Inferences were unchanged in the per-protocol and sensitivity analyses. Change in MELD score was not affected by either previous consumption of alcohol (appendix p 10) or length of abstinence from alcohol (appendix p 11). We also evaluated MELD and UKELD scores at days 180 and 360 (appendix p 17), we detected no differences between groups.

**Table 2:** Circulating leucocyte and haematopoietic stem-cell numbers before and after G-CSF

	**G-CSF only (n=26)**			**G-CSF plus CD133-positive cell infusion (n=28)**		
	Before G-CSF	After G-CSF	After to before ratio	Before G-CSF	After G-CSF	After to before ratio
White cell count (per μL)	3715·0 (2920·0–4955·0)	25 605·0 (21 610·0–33 000·0)	6·8 (4·5–7·7)	4000·0 (3180·0–5780·0)	24 600·0 (18 590·0–35 150·0)	5·7 (4·4–8·3)
CD34 cell count (per μL)	2·9 (1·5–4·7)	28·8 (16·9–50·9)	8·7 (7·0–16·5)	2·4 (1·5–3·3)	31·4 (20·4–36·8)	10·7 (6·0–22·7)
CD133 cell count (per μL)	2·8 (1·6–5·0)	24·7 (14·4–39·3)	8·1 (5·0–21·4)	2·4 (1·1–4·1)	21·7 (16·9–32·2)	13·8 (4·5–23·0)
CD133-positive CD45^low^ cells per μL	1·2 (0·5–2·1)	18·7 (10·9–27·1)	15·4 (9·9–24·5)	0·8 (0·5–1·8)	18·8 (13·2–31·9)	24·6 (5·6–60·0)
Proportion of CD133-positive cells of viable CD45-positive cells	0·1% (0·0–0·1)	0·1% (0·1–0·1)	1·2% (0·9–3·0)	0·1% (0·0–0·1)	0·1% (0·1–0·1)	1·5% (0·7–4·5)
Proportion of CD133-positive CD45^low^ cells of viable CD45-positive cells	0·0% (0·0–0·1)	0·1% (0·0–0·1)	2·1% (1·1–4·1)	0·0% (0·0–0·1)	0·1% (0·1–0·1)	3·1% (1·0–6·8)

Data are median (IQR). G-CSF=granulocyte colony-stimulating factor.

Patients in the treatment groups who received G-CSF had increases in the number of circulating white blood cells, and in CD34-positive and CD133-positive cell populations (table 3). Data on the number of HSCs in the apheresis and post-isolation cell product are shown in the appendix (p 14).

**Table 3:** Change in liver parameters from baseline to day 30 and day 90

	**Standard care (n=27)**		**G-CSF only (n=26)**		**G-CSF plus CD133-positive cell infusion (n=28)**	
	Day 30	Day 90	Day 30	Day 90	Day 30	Day 90
**Liver disease severity**						
MELD score	−0·3 (−0·8 to 0·2)	−0·5 (−1·5 to 1·1)	0·0 (−1·0 to 1·0)	−0·5 (−1·7 to 0·5)	−0·1 (−2·2 to 1·0)	−0·5 (−1·3 to 1·0)
UKELD score	−1·0 (−2·2 to 0·4)	−0·1 (−3·2 to 1·7)	−0·3 (−1·1 to 1·0)	0·5 (−1·0 to 1·4)	−1·0 (−1·9 to −0·2)	−0·5 (−1·2 to 0·5)
Child-Pugh score	0·0 (0·0 to 0·0)	0·0 (0·0 to 0·0)	0·0 (0·0 to 1·0)	0·0 (0·0 to 1·0)	0·0 (−1·0 to 1·0)	0·0 (0·0 to 1·0)
**Full blood count**						
Haemoglobin (g/dL)	−0·1 (−0·7 to 0·4)	0·3 (−0·6 to 0·5)	−1·0 (−1·4 to −0·4)*	−0·3 (−0·6 to 0·5)	−1·0 (−1·6 to −0·4)†	−0·4 (−1·0 to 0·4)
White blood cells (× 10^9^ per L)	0·4 (−0·3 to 1·0)	0·3 (−0·2 to 0·6)	−0·7 (−1·1 to −0·2)‡	−0·1 (−0·8 to 0·6)	−1·0 (−1·6 to −0·3)§	−0·2 (−1·1 to 0·5)
Platelets (× 10^9^ per L)	2·0 (−7·0 to 9·0)	−0·5 (−8·0 to 9·0)	13·0 (2·0 to 23·5)¶	0·0 (−8·0 to 7·0)	8·0 (−4·0 to 30·0)	1·0 (−3·0 to 5·0)
**Biochemistry**						
Sodium (mmol/L)	1·0 (−1·0 to 4·0)	0·0 (−2·0 to 3·0)	0·0 (−1·0 to 2·0)	−1·0 (−2·0 to 1·0)	1·0 (−1·0 to 3·0)	0·0 (−1·0 to 3·0)
Potassium (mmol/L)	0·0 (−0·2 to 0·2)	0·1 (−0·1 to 0·3)	0·0 (−0·2 to 0·3)	0·2 (−0·2 to 0·5)	−0·1 (−0·3 to 0·2)	−0·1 (−0·3 to 0·3)
Urea (mmol/L)	0·3 (−0·1 to 0·9)	0·1 (−0·4 to 1·0)	0·0 (−0·5 to 0·7)	0·2 (−0·6 to 0·9)	0·2 (−0·6 to 0·5)	−0·1 (−1·0 to 0·4)
Creatinine (μmol/L)	3·0 (−2·0 to 5·0)	1·0 (−2·0 to 10·0)	2·0 (−5·0 to 4·0)	2·5 (−1·0 to 10·0)	1·0 (−3·0 to 8·0)	1·5 (−4·0 to 9·0)
**Liver function tests**						
Total bilirubin (μmol/L)	−5·0 (−9·0 to 3·0)	−5·0 (−12·0 to 3·0)	−2·0 (−10·0 to 8·0)	−6·0 (−12·0 to −1·0)	−2·5 (−9·5 to 10·0)	−2·0 (−9·0 to 7·0)
Albumin (g/L)	0·0 (−3·0 to 1·0)	0·0 (−3·0 to 1·0)	−2·0 (−3·0 to −1·0)	−2·0 (−3·0 to 0·0)	−3·0 (−4·0 to −1·0)	−1·0 (−4·0 to 2·0)
International normalised ratio	0·0 (0·0 to 0·0)	0·0 (−0·1 to 0·1)	0·0 (−0·1 to 0·1)	0·0 (−0·1 to 0·1)	0·0 (−0·1 to 0·1)	0·0 (0·0 to 0·0)
Alanine aminotransferase (IU/L)	1·0 (−4·0 to 3·0)	0·0 (−4·0 to 4·0)	−3·0 (−7·0 to 1·0)	−2·0 (−9·0 to 4·0)	−2·5 (−5·0 to 0·0)	−4·5 (−8·0 to 0·0)
Aspartate aminotransferase (IU/L)	−0·5 (−6·0 to 5·0)	−2·0 (−6·0 to 4·0)	−3·0 (−8·0 to 2·0)	−4·0 (−13·5 to 4·0)	−3·0 (−9·0 to 1·0)	−2·0 (−9·0 to 1·0)
γ glutamyl transferase (IU/L)	−1·0 (−7·0 to 3·0)	−1·0 (−7·0 to 3·0)	−8·0 (−22·5 to 0·0)	−10·0 (−42·5 to 0·5)	−5·0 (−22·0 to 1·0)	−3·0 (−18·0 to 1·0)
Alkaline phosphatase (IU/L)	−8·0 (−28·0 to 14·0)	−4·0 (−20·0 to 11·0)	7·0 (−8·0 to 21·0)	−5·0 (−16·0 to 7·0)	8·0 (0·0 to 11·0)	1·5 (−10·0 to 24·0)
α fetoprotein (AFP)	0·0 (0·0 to 0·0)	0·0 (−1·0 to 1·0)	0·0 (−1·0 to 0·0)	0·0 (−1·0 to 0·0)	0·0 (−0·5 to 0·0)	0·0 (−1·0 to 0·0)
**Non-invasive hepatic biomarkers**						
Enhanced Liver Fibrosis (ELF)	··	−0·1 (−0·4 to 0·6)	··	0·0 (−0·4 to 1·0)	··	0·1 (−0·6 to 0·5)
Fibroscan	··	0·0 (−1·6 to 8·6)	··	0·0 (−11·9 to 9·5)	··	0·5 (−3·8 to 10·1)
**Quality of life**						
Overall CLDQ score	··	0·2 (−0·1 to 0·6)	··	−0·1 (−0·4 to 0·3)	··	0·0 (−0·2 to 0·2)

Values are change in medians (IQR). Statistical comparisons were made between change at day 30 and 90 to baseline between the treatment and control groups, and unless indicated, there were no significant differences. G-CSF=granulocyte colony-stimulating factor. UKELD=UK End-Stage Liver Disease Score. CLDQ=Chronic Liver Disease Questionnaire.

* p=0·0017.

† p=0·0006.

‡ p=0·0024.

§ p=0·0002.

¶ p=0·0053.

Neither treatment group had a reduction in mortality or admissions to hospital compared with the control. Patients in both treatment groups had to be considered for liver transplantation, and patients in the G-CSF plus stem-cell infusion group had more serious adverse events (12 [43%] of 28 patients) than patients in the G-CSF group (three [11%] of 28 patients) and the standard care group (three [12%] of 26 patients; table 4). At the time of adjudication, none of the serious adverse events were judged to be related to treatment. The frequency of adverse events reported by CTCAE category and grade was similar between groups, except for nervous system disorders and musculoskeletal disorders, which were more common in the treatment groups than in the standard care group (appendix p 18). Patients were monitored for a year, including standard screening for hepatocellular carcinoma every 6 months and no malignancies were detected. We also followed up patients for outcomes such as death, assessment for liver transplantation, and serious adverse events up to 1 year after randomisation (table 4). Three patients died during the study, including one in the standard care group (variceal bleed) and two in the G-CSF plus stem-cell infusion group (one myocardial infarction and one progressive liver disease).

**Table 4:** Adverse events

	**Standard care**	**G-CSF only**	**G-CSF plus CD133-positive cell infusion**
**Deaths**			
Event 1	Variceal bleed (2·3 months)	··	Myocardial infarction (3·9 months)
Event 2	··	··	Progressive liver disease (8·9 months)
**Assessed for liver transplant**			
Event 1	Assessed due to decompensation (7·9 months); not listed due to ventricular tachycardias on electrocardiogram	Assessed and listed due to decompensation (1·7 months); transplanted (4·3 months)	Assessed and listed due to decompensation (3·9 months); transplanted (10·2 months)
Event 2	··	Assessed and listed due to decompensation (4·9 months); transplanted (16·9 months)	Assessed and listed due to decompensation (10·0 months); transplanted (13·6 months)
Event 3	··	Assessed and listed due to poor synthetic function (5·1 months); listed but removed due to improvement	Assessed and listed due to decompensation (10·5 months); not transplanted
Event 4	··	Assessed and listed due to decompensation (12·0 months); transplanted (15·0 months)	··
**Serious adverse events**			
Event 1	Hypoglycaemia (2·4 months)*	Oesophageal variceal bleed (2·1 months)*	Diarrhoea and pulmonary sepsis (0·8 months)
Event 2	Hepatic decompensation (2·3 months) and later died	Urinary retention and ascites (1·7 months)*	Sepsis and encephalopathy (2·3 months);* acute kidney injury (2·8 months)*
Event 3	··	Hepatic decompensation and ascites (3·0 months)*	Cardiac failure (5·8 months)*
Event 4	··	··	Ascites and encephalopathy (0·7 months);* ascites and encephalopathy (2·3 months)*
Event 5	··	··	Abdominal sepsis (0·3 months)*
Event 6	··	··	Peripheral oedema (1·7 months)*
Event 7	··	··	Sepsis and ascites (1·6 months)*
Event 8	··	··	Encephalopathy (2·0 months)*

Unless indicated otherwise, events represent different patients within the study. G-CSF=granulocyte colony-stimulating factor.

* Resolved with no sequelae.

## Discussion

In this study, we found that neither G-CSF nor G-CSF plus stem-cell infusion showed improvement in MELD score in patients with compensated liver cirrhosis. These results contrast with the findings of previous smaller studies.^8^

We chose MELD score as the primary endpoint because it is an objective prognostic scoring system validated in large independent cohorts worldwide for patients with chronic liver disease. It independently predicts clinical decompensation in patients with compensated cirrhosis^15^ and is highly accurate in the prediction of 1-week, 3-month, and 1-year mortality. For any given MELD score, the magnitude and direction of change in score during the previous 30 days is a significant independent predictor of mortality, making change in score a more important determinant than initial MELD score alone. The magnitude of the change in MELD score can also predict the development of complications of cirrhosis such as ascites and variceal bleeding. We supplemented the use of MELD as a primary endpoint with many secondary endpoints of liver dysfunction, as well as markers of liver fibrosis (serum ELF and Fibroscan), none of which improved with therapy.

We specifically chose to recruit patients with predominantly compensated cirrhosis, as opposed to those with more advanced decompensation, in the belief that the included patients had a greater potential for regeneration and regression of fibrosis. In this respect, a tangible effect of stem-cell therapy could have been to prevent disease progression that would require consideration for liver transplantation. It seems highly unlikely that the interventions in this study would be more effective in a cohort of patients with more advanced decompensated disease given that the fibrosis in such patients would be more advanced and less likely to be reversible.^16^

This study has several strengths. It is, to our knowledge, the largest and most rigorous randomised controlled trial on stem-cell therapy for cirrhosis of the liver thus far, with robust clinical trial governance and sufficient power to detect a clinically meaningful difference. The trial also included a representative spread of causes of liver disease, between which we saw no differences in response to the interventions (appendix p 12). Moreover, the patients were well characterised within a narrow range of MELD scores (11·50–15·50) and with respect to potentially confounding effects such as the duration of abstinence from alcohol and concomitant use of antiviral therapy. The study also used a defined stem-cell population, which is important for regulatory and scientific reasons; in animal models, mixed bone marrow infusions have been reported to contribute to liver fibrosis,^4^ whereas purified HSCs reduce fibrosis.^6^ CD133-positive cells represent a more enriched subpopulation of the CD34-positive cells, with true pluripotent stem cells constituting about 0·1% of a CD133-positive population.^17^ Moreover CD133-selected cells have previously been infused in patients with liver^9^ and cardiovascular disease,^18^ producing benefits and with no safety concerns. We chose the dose used in this study (0·2 × 10^6^ cells per kg per infusion) because it was higher than that used in previous studies and we felt it to be achievable. Notably, in several patients, this amount of cells could not be mobilised, suggesting that our chosen number of cells might have been too high. CD133-positive doses of up to 2·5 × 10^6^ cells per kg have been used during bone marrow transplantation, although this was in the setting of bone marrow malignancy and chemotherapy.

Another strength of this study was the inclusion of a stand-alone G-CSF group. Results from a dose-finding study have showed that G-CSF at a dose of 15 μg/kg per day for 5 days was safe in patients with cirrhosis and was deemed to be the optimum dose for effective stem-cell mobilisation without adverse effects.^19^ This dose is higher than the routinely used dose of 10 μg/kg per day because patients with cirrhosis have a poorer mobilisation capacity than other patients, which is possibly related to splenic sequestration and poor bone marrow function. This dose was well tolerated by patients and no adverse effects were recorded. Another previous study that used the standard dosage showed a reversible increase in spleen size.^20^ While G-CSF is used to mobilise cells for leukapheresis, it has also been reported to improve liver regeneration and function in preclinical studies^21^ and to improve mortality in a clinical trial of acute-on-chronic liver failure in India, possibly through a reduction in deaths from sepsis.^22^ Although differing in dose and clinical target from the study by Garg and colleagues,^22^ our study found no evidence of any benefit for the use of G-CSF in terms of liver fibrosis or function, or indeed sepsis, as has previously been reported.

A potential limitation of the study could be the absence of a true placebo and masking of treatment allocation, although in the absence of an effect this point is less pertinent. Given the overt side-effects of G-CSF (eg, bone pain), a placebo would be readily unmasked, whereas sham leukapheresis would pose ethical challenges.

Another limitation is the absence of a histological endpoint. Although histological analysis to assess the effect on fibrosis and hepatocyte proliferation might have been revealing, we considered this to be unlikely. Microscopic analysis of needle biopsy material in patients with cirrhosis can be misleading because of the macrohistological nature of the disease. For example, biopsy of regenerative nodules surrounded by bands of fibrosis can have the appearance of normal liver, and different stages of fibrosis can be seen when multiple biopsies are taken from the same liver at transplantation.^23^ Furthermore, in the setting of cirrhosis and coagulopathy, two biopsies within a short timeframe would constitute a substantial undertaking not without clinical risk; even transjugular liver biopsy in patients with cirrhosis has major complications (eg, perforation of the hepatic capsule, cholangitis, and intraperitoneal bleeding) in 1–3% of cases. Mortality related to the procedure varies from 0·2% to 0·3%.^24^ Indeed, it is not clear that regulatory approval would even be granted for such a study in western Europe or the USA. Finally, this trial was powered on a clinically relevant marker of liver dysfunction, for which a liver biopsy would provide no relevant information. It is possible that the absence of histological confirmation of cirrhosis resulted in patients without cirrhosis being included, although this is very unlikely given that impaired liver synthetic function was a key inclusion criteria.

We decided not to track cells after infusion to confirm homing to the liver. Although these results would have been informative, the technical and regulatory barriers to non-invasively labelling and tracking such cells are substantial and there is a substantial risk that labelling of CD133-positive cells before their infusion could alter their viability and efficacy. While our published preclinical data^6^ suggested that HSCs efficiently engraft the recipient liver at first, there is minimal medium-term engraftment, and, notably, the HSCs induce longer-lasting changes in endogenous inflammatory cells such as macrophages.^6^ Clinical data from the setting of alcoholic hepatitis also support this possibility.^25^

The absence of any effect of HSC infusions on liver fibrosis and function in this study differs from the positive effects reported by us and others in rodent models of liver fibrosis.^6^ This result comes despite the dose of cells and G-CSF being higher than that reported in other studies.^8^ Furthermore, given the failure to mobilise sufficient HSCs in all patients in this study, it would be difficult to aim for an even higher infused cell dose. Thus, this result is likely to be a true reflection of the lack of action of HSCs in the setting of advanced liver fibrosis and suggests caution when extrapolating the efficacy of any putative antifibrotic or proregenerative therapy in rodent models of fibrosis to the clinical setting. Fibrosis is qualitatively and quantitatively different in rodent models, with rodent fibrosis having less collagen cross-linking and more spontaneous remodelling than is typically seen in humans.^26^, ^27^ Studies in rodents also often use freshly isolated cells derived from healthy animals, thus in this study, the use of autologous frozen cells might be relevant, although frozen cells are commonly used in bone marrow transplantation to good effect.

It is possible that the peripheral venous administration of the cells resulted in few cells reaching the liver, and in theory, direct administration of cells into the vessels supplying the liver (the portal vein and hepatic artery) might be more effective. However, this approach is invasive and carries substantial risks, including bleeding and thrombosis. Although such approaches were initially reported as being used without complications, a later study^28^ showed that direct infusions can lead to portal hypertensive bleeding following cell injection and another study^29^ was terminated early following complications in two of four patients after hepatic artery injection.^29^ Moreover, the scientific literature does not support any advantage of directed infusion of cells to the liver over peripheral infusion.^30^

Our trial found no evidence to support any benefit from G-CSF alone or G-CSF plus stem-cell infusion in liver cirrhosis. Given the often intense enthusiasm surrounding stem-cell therapy, despite potential safety concerns,^31^ this important negative result reinforces the need for rigorous clinical trials to test efficacy in the field of stem cell-based therapy. This study also provides a template for future studies of novel anti-fibrotic therapies in patients with liver cirrhosis.
