# Supplementary material for: Granulocyte colony-stimulating factor and autologous CD133-positive stem-cell therapy in liver cirrhosis (REALISTIC): an open-label, randomised, controlled phase 2 trial
Source: Lancet Gastroenterol Hepatol. 2017 Nov 7;3(1):25–36. doi: 10.1016/S2468-1253(17)30326-6 (PMC5738975; doi:10.1016/S2468-1253(17)30326-6)
Supplement: Supplementary appendix [file mmc1.pdf]

THE LANCET
Gastroenterology & Hepatology

Supplementary appendix

This appendix formed part of the original submission and has been peer reviewed. We post it as supplied by the authors.

Supplement to: Newsome PN, Fox R, King AL, et al. Granulocyte colony-stimulating factor and autologous CD133-positive stem-cell therapy in liver cirrhosis (REALISTIC): an open-label, randomised, controlled phase 2 trial. *Lancet Gastroenterol Hepatol* 2017; published online Nov 7. http://dx.doi.org/10.1016/S2468-1253(17)30326-6.

Supplementary Appendix

TABLE OF CONTENTS **Page**

1. List of investigators
1.1 Other members of the REALISTIC trial team **2**
1.2 Members of the REALISTIC Data Management Committee **2**

2. Supplementary Methods
2.1 Inclusion and exclusion criteria in full **3**
2.2 Trial schedule and data collection **7**
2.3 Additional analysis – statistical methodology (Incl **Table S1**) **9**
2.4 Additional analysis – effect of alcohol abstinence (Incl **Tables S2/3/4**) **13**

3. Figures/Tables
Table S5: Dose of GCSF and $CD133^+$ cells administered to Arm 3 patients **17**
Table S6: Cellular content of apheresis and post-MACS selection product **19**
Figure S1: Waterfall plot of day 90 change in MELD **20**
Figure S2: Waterfall plot of best change in MELD (to day 90) **21**
Table S7: Change in MELD and UKELD from baseline to D180 and D360 **22**
Table S8: Adverse event table **23**

1. List of investigators

1.1 Other members of the REALISTIC trial team:

Queen Elizabeth University Hospital Birmingham/NIHR Liver BRU/CRUKCTU (Birmingham, UK): Somto Eruchie, Salma Iqbal, Jennifer Keely, Michelle Yun Kyong Lee, Christina Russell, Manpreet Wilkhu and nursing staff at the WTCRF.

Nottingham University Hospitals NHS Trust/ Nottingham Digestive Diseases BRU (Nottingham, UK): Maggie Nicholls and Susanne Henry.

1.2 Members of the REALISTIC Data Management Committee (DMC):

Professor David Jones (DMC Chair, Independent Liver expert), Professor of Hepatology, Newcastle, UK.
Dr Sarah Brown (Independent Senior Statistician), Principal statistician, Clinical Trials Research Unit, Leeds Institute of Clinical Trials Research, University of Leeds, Leeds, UK.
Professor Mark Thursz (Independent Liver expert), Consultant in Gastroenterology and Hepatology, Obesity Physician, Guy's & St Thomas' Hospital/Royal Free Hospital, London, UK.

2. Supplementary Methods

2.1 Inclusion and exclusion criteria in full

Patients were eligible for the trial if aged 18–70 with compensated cirrhosis and a Model for End-stage Liver Disease (MELD) score greater than or equal to 11.5 and
less than 15.5. The specific inclusion criteria are listed below. In addition to the general exclusion criteria there were specific exclusion criteria related to liver disease and to the safety of GCSF administration, which are listed below.

Inclusion Criteria

1. 18< Age ≤75 at randomisation
2. 11.00 < MELD <15.50 at randomisation
3. Aetiology of liver disease, one or more of:
   - Alcohol related Liver Disease
     - Features (clinical, biochemical, histological or radiological) of chronic liver disease with a compatible history of alcohol excess (>80g/day), in the absence of other causes of chronic liver disease
     - Abstinent >6 months prior to enrolment
   - Hepatitis C
     - Positive HCV Antibody
     - Not currently on antiviral therapy
   - Hepatitis B
     - Positive HBsAg and Anti-HBc
     - Established on antiviral therapy with adequate viral suppression
   - Primary Biliary Cirrhosis
     - 2 out of Cholestatic LFTs
     - Positive AMA (>1:40)
     - Compatible Histology
     - If already receiving Ursodeoxycholic Acid: must be established on current dose >3 months prior to enrolment
   - Haemochromatosis
     - Diagnosis made on basis of compatible Biochemistry (Transferrin Sat >60%, Ferritin >400), Genotype (Homozygous C282Y or H63D, Compound Heterozygote) or Histology
   - Cryptogenic cirrhosis
     - Diagnosis of cirrhosis unattributable to any other cause
   - Non Alcoholic Fatty Liver Disease (NAFLD)
     - Either: Histological evidence of steatosis in the absence of other liver diseases
     - Or: Imaging compatible with NAFLD (eg Fatty infiltration of liver) and one or more risk factors (e.g. elevated BMI, T2DM, Hypertriglyceridaemia, Hypertension)
     - And: The absence of significant alcohol consumption (<20g/day) and no evidence of other causes of chronic liver disease
   - Alpha-1 Antitrypsin Deficiency
     - Diagnosis based on compatible genetic, phenotypic or histological testing.
4. Cirrhosis, defined as one of:

   - Previous Liver Biopsy confirming histological features of cirrhosis
   - Transient Elastography (Fibroscan) > 18 kPa
   - Clinical and Radiological features that in the opinion of the investigator correlate with a diagnosis of cirrhosis
   - AST:Platelet Ratio Index (APRI) > 2.0 (APRI = (([AST]/ULN)*[Plt])x100)

Exclusion Criteria

- Refusal or inability to give informed consent to participate in the study
- Average alcohol ingestion >21 units/week (male) / >14units/week (female)
- Other cause of chronic liver disease / cirrhosis not included in listed aetiologies – this is left to the clinical judgement of the investigator based on previous investigations and trial screening.
- Ascites Unless, in the opinion of the investigator, the ascites is minimal and well controlled with no changes to diuretic therapy in last 3 months
- Encephalopathy Current or requiring hospitalisation for treatment in last 3 months.
- Portal Hypertensive Bleeding Active episode of bleeding requiring treatment or Hospitalisation in the last 3 months
- Hepatocellular Carcinoma – uncertain cases to be discussed at local Hepatobiliary Multidisciplinary meeting, Dysplastic or Indeterminate nodules to be excluded, Regenerative or other nodules to be included at discretion of MDM
- Previous diagnosis of Hepatocellular Carcinoma
- Previous Liver Transplant
- Listed for Liver Transplantation
- Recent history of pulmonary infiltrates or pneumonia: patients should have completely recovered from any previous episodes, both clinically and radiologically.
- Any situation that in the Investigators opinion may interfere with optimal study participation such as alcohol or drug abuse, domicile too distant from study site, potential non-compliance or inability to co-operate
- **Presence of other clinical problems (cardiovascular, pulmonary, GI, renal, metabolic haematological, neurological, psychiatric, systemic, ocular, gynaecological or infectious deemed to potentially compromise patient safety)**
- **Presence of cancer in last 5 years**
- **Pregnancy or breastfeeding.**

2.2 Trial schedule and data collection

	Screening	Treatment					Follow Up	
	Visit 1 (screening)[1,2]	Visit 2a – 2e[1] (1st – 5th Treatment Day)[7]	Visit 2f (5th/6th Treatment Day)[7]	Visit 3 (Day 30)	Visit 4 (Day 60)	Visit 5 (Day 90)	Visit 6 (Day 180)	Visit 7 (Day 360)
Informed Consent	X							
Clinical Assessment[2]	X	X	X	X	X	X	X	X
Vital Signs[3]	X	X	X	X	X	X	X	X
Screening Blood Tests[4]	X							
ECG	X							
Standard Blood Tests[5]	X	X	X	X	X	X	X	X
Mandaory Microbiology[7]	X							
Abdominal USS	X		X				X	
Fibroscan	X					X	X	X
ELF Panel	X			X	X	X	X	X
CLDQ	X					X	X	X
G-CSF Administration		GROUP 2<br>GROUP 3						
Leukopheresis			GROUP 3					
Blood Test for Circ CD34+		GROUP 2<br>GROUP 3	GROUP 2<br>GROUP 3					
Blood Test for Circ CD133+		GROUP 2<br>GROUP 3	GROUP 2<br>GROUP 3					
CD133+ Cell Infusion			GROUP 3[8]	GROUP 3	GROUP 3			
Adverse Effects[6]	X	X	X	X	X	X	X	X
Clinical Events[6]		X	X	X	X	X	X	X
Concomitant Medication	X	X	X	X	X	X	X	X

[1] For patients in arms 1 and 2 Visit 1 and visit 2a should be combined into one day where possible. For patients in arm 3, timing of visit 2a will depend on scheduling of leukapheresis. [2] All screening tests must be completed less than 7 days prior to randomisation and treatment and must start less than 7 days following randomisation. Day of randomisation will be considered as Day 1 for scheduling purposes. [3] Clinical assessment consists of complete history and examination at screening and focussed history and relevant examination at subsequent visits. [4] Vital Signs to include heart rate, blood pressure, temperature and weight. [5] Screening blood tests as detailed in protocol

2.3 Additional analysis - statistical methodology

A model incorporating splines was constructed to assess fit. Model selection was then performed beginning with a mixed-model including just treatment arm (factor) and time (continuous) covariates, and then by iteratively increasing flexibility as required to find the most parsimonious model resulting in approximately optimum fit; polynomial, interaction, and change-point terms were all explored in doing so.

The study was designed to detected differences in primary 1 only and not to detect differences in trend or interactions, which was proposed in order to explore the change in longitudinal outcome measures over the study period. As such, less emphasis is placed upon p values for co-primary 2; rather a focus on magnitude of trend.

2.3.1 Co-primary 2 results

What follows describes the results of the final mixed effects model with a change-point for co-primary 2. Note that in a departure from the convention adopted thus far in this supplement rate of change is described in per week since resulting estimates are more tangible in the context of generally small changes.

$$
\begin{aligned}
\mathrm{Y}_{ij} = \beta_0 + \beta_1 \mathrm{m}_{2i} + \beta_2 \mathrm{m}_{3i} + \beta_3 \mathrm{t}_{ij1} + \beta_4 \mathrm{t}_{ij2} + \\
\beta_5 \mathrm{m}_{2i}\mathrm{t}_{ij1} + \beta_6 \mathrm{m}_{3i}\mathrm{t}_{ij1} + \beta_7 \mathrm{m}_{2i}\mathrm{t}_{ij2} + \beta_8 \mathrm{m}_{3i}\mathrm{t}_{ij2} \\
+\mathrm{b}_i + \varepsilon_{ij}, \qquad \varepsilon_{ij} \sim N(0, \sigma^2)
\end{aligned}
$$

where

- Y_{ij} is the MELD score for patient i with four planned measurements at time points j at 0 and approximately 4.3, 8.6 and 12.9 weeks (corresponding to visit days 30, 60 and 90)
- m_{2i} and m_{3i} are dummy variables for treatment groups 2 and 3 respectively
- t_{ij} is time (in weeks) ranging from 0 to 12.9 weeks (90 days) for patient i
- $\mathrm{t}_{ij1} = \mathrm{t}_{ij} - 4.3, \mathrm{t}_{ij}$ for ϵ $[0, 4.3]$, and 0 otherwise, and represents up to 4.3 weeks
- $\mathrm{t}_{ij2} = \mathrm{t}_{ij} - 4.3, \mathrm{t}_{ij}$ for ϵ $[4.3, 12.9]$, and 0 otherwise, and represents from 4.3 weeks
- b_i denotes the patient-specific random intercept, $\mathrm{b}_i \sim N(0, \sigma_b^2)$
- β_n, for $n \epsilon [1, 8]$, represent coefficients of the fixed effect parameters.

The continuous time-scale, representing timing of measurement, was split at 4.3 weeks (30 days) allowing differing trends to be explored both prior to and after this point, hereafter referred to as period 1 and 2 respectively. Splits at 8.6 weeks, and +/- 5 days either side of the particular change-point were also explored but found to fit the data less well. The model also incorporates interactions between time period and group, to allow for the rate of change to differ between groups.

The estimated coefficients of time for period 1 (-0.043) and period 2 (0.005) can be considered the average rate of change in MELD (per week) in group 1, and indicate a small reduction in MELD in period 1, and an approximately constant thereafter. In neither case was there evidence that the rates differed from zero (p=0. 64 and 0. 91 respectively). Interaction terms estimated the difference in rate of change for each treatment group relative to group 1. In period 1 the group 2 and 3 interaction estimates of 0.14 (p=0. 28) and 0.022 (p=0. 87) were not found to differ from zero, and hence there was no evidence that the rates of change differed from group 1. When combined (additively) with corresponding time estimates, the interaction terms for period 1 can be interpreted on average as an increment of 0.093 for group 2, and a decrement of -0.021 for group 3 for each unit increase in time, i.e. a week.

Similarly, there was no evidence of non-zero estimates in period 2, with group 2 and 3 with interaction coefficients -0.066 (p=0.31) and -0.005 (p=0. 94) respectively. In combination the estimates indicated a weekly decrement of 0.071 for group 2, and an approximately zero change for group 3. Hence there was no evidence that the rates in period 2 differed from group 1. Furthermore, testing for a change-point indicated that the trends do not differ between periods with p=0.76. Of note the corresponding model term does not belong to the final model described in the foregoing.

Table S1: Mixed model for MELD over 4.3 weeks (90 days).

**Parameter**	**Estimate**		**p-value**	**95% confidence interval**
**Treatment**				
group 2	β_1	0.15	0.76	-0.85, 1.16
group 3	β_2	0.13	0.81	-0.93, 1.18
period 1 (weeks 0 to 4.3)	β_3	-0.043	0.64	-0.22, 0.14
period 2 (week 4.3 to 12.9)	β_4	-0.005	0.91	-0.099, 0.088
**Interaction terms**				
period 1 & group2	β_5	0.14	0.28	-0.11, 0.38
period 1 & group3	β_6	0.022	0.87	-0.24, 0.28
period 2 & group2	β_7	-0.066	0.31	-0.192, 0.060
period 2 & group3	β_8	0.005	0.94	-0.13, 0.136
constant	β_0	12.98	<0.0001	12.26, 13.71

Legend for table S1: Mixed model for MELD over 4.3 weeks (90 days). Incorporates change point at 4.3 weeks (day 30), and allows for changes in trend to differ between groups before and after change-point.

<u>2.4 Additional analysis – effect of alcohol abstinence</u>

An unplanned analyses explored the impact of alcohol history on change in MELD score. The final MELD mixed model was adjusted for 'ever' versus 'never' drinkers, and the final model was also fitted in only those patients that had ever drank, with adjustment for length of abstinence.

Results

There was no evidence that having previously consumed alcohol affected MELD (p=0.26) (Appendix **Table S2**). Nor was there evidence that length of abstinence in those patients that had consumed alcohol had any impact on MELD (p=0.41) (Appendix **Table S3**).

Table S2: Final mixed model adjusted for alcohol consumption.

**Parameter**	**Estimate**		**p-value**	**95% confidence interval**
**Treatment**				
group 2	β_1	-0.43	0.46	-1.57, -1.27
group 3	β_2	-0.12	0.84	-1.27, -0.19
period 1 (weeks 0 to 4.3)	β_3	0.0016	0.99	-0.19, -0.28
period 2 (week 4.3 to 12.9)	β_4	-0.0049	0.97	-0.28, 0.00
**Interaction terms**				0.00, -0.24
period 1 & group2	β_5	0.029	0.83	-0.24, -0.24
period 1 & group3	β_6	0.032	0.82	-0.24, -0.43
period 2 & group2	β_7	-0.066	0.72	-0.43, -0.45
period 2 & group3	β_8	-0.075	0.70	-0.45, 0.00
**Alcohol**				0.00, 0.00
Have consumed alcohol	β_9	0.455695	0.26	0.00, 11.86
constant	β_0	12.8448	<0.0001	11.86, 0.00

Legend for Table S2: final mixed model adjusted for alcohol consumption. With parameters as before and with β_9 the coefficient for 'having consumed alcohol (versus not).

Table S3: Final mixed model adjusted for length of abstinence

Parameter	Estimate		p-value	95% confidence interval
**Treatment**				
group 2	β_1	-0.224	0.77	-1.75, 1.30
group 3	β_2	0.074	0.92	-1.42, 1.57
period 1 (weeks 0 to 4.3)	β_3	0.0015	0.99	-0.25, 0.25
period 2 (week 4.3 to 12.9)	β_4	-0.012	0.95	-0.36, 0.34
**Interaction terms**				
period 1 & group2	β_5	0.065	0.72	-0.29, 0.42
period 1 & group3	β_6	0.12	0.51	-0.24, 0.48
period 2 & group2	β_7	-0.069	0.79	-0.56, 0.43
period 2 & group3	β_8	-0.17	0.49	-0.66, 0.31
**Alcohol**				
Length of abstinence (months)	β_9	0.0047	0.41	-0.01, 0.02
constant	β_0	12.84	<0.0001	11.65, 14.03

Legend for Table S3: final mixed model adjusted for length of abstinence. With parameters as before and with β_9 the coefficient for length of abstinence in months.

Table S4: Final mixed model aetiology

Parameter	Estimate		p-value	95% confidence interval
**Treatment**				
group 2	β_1	0.14	0.78	-0.86, 1.14
group 3	β_2	0.11	0.83	-0.94, 1.16
period 1 (weeks 0 to 4.3)	β_3	-0.044	0.63	-0.22, 0.13
period 2 (week 4.3 to 12.9)	β_4	-0.0064	0.89	-0.10, 0.087
**Interaction terms**				
period 1 & group2	β_5	0.14	0.28	-0.11, 0.38
period 1 & group3	β_6	0.023	0.86	-0.24, 0.28
period 2 & group2	β_7	-0.065	0.31	-0.19, 0.061
period 2 & group3	β_8	0.0061	0.93	-0.13, 0.14
**Aetiology**				
Hep-C	β_9	-0.55	0.28	-1.56, 0.45
Other	β_{10}	-0.0036	0.99	-0.67, 0.66
constant	β_0	13.06	<0.0001	12.26, 13.86

Legend for table S4: final mixed model aetiology. With parameters as before and with β_9 and β_{10} the respective coefficients of aetiology levels (versus alcohol related).

3. Figures/Tables

Table S5: Dose of GCSF and CD133$^+$ cells administered to Arm 3 patients

Patient number	GCSF dose (mcg/kg)					Cell dose administered (10^6 cells /kg)				
	**Day 1**	**Day 2**	**Day 3**	**Day 4**	**Day 5**	**Dose 1**	**Dose 2**	**Dose 3**	Modified ITT analysis	Per Protocol analysis
1	15.00	15.00	15.00	15.00	15.00	0.20	0.20	0.20	1	1
2	15.00	15.00	15.00	15.00	15.00	0.20	0.20	0.20	1	1
3	.	.	.	.	.	.	.	.	0	0
4	15.00	15.00	15.00	15.00	15.00	0.20	0.14	0.19	1	1
5	15.00	15.00	15.00	15.00	15.00	0.20	0.20	0.20	1	1
6	15.00	15.00	15.00	15.00	15.00	0.20	0.20	0.20	1	1
7	15.00	15.00	15.00	15.00	15.00	0.17	.	0.17	1	0
8	15.00	15.00	15.00	15.00	15.00	0.20	0.16	0.20	1	1
9	15.00	15.00	15.00	15.00	15.00	0.21	0.21	0.21	1	1
10	15.00	15.00	15.00	15.00	15.00	0.20	0.20	0.20	1	1
11	15.00	15.00	15.00	15.00	15.00	0.20	.	.	1	0
12	15.00	15.00	15.00	15.00	15.00	0.20	0.20	0.20	1	1
13	15.00	15.00	15.00	15.00	15.00	0.19	0.19	0.19	1	1
14	15.00	15.00	15.00	15.00	15.00	0.20	0.20	0.20	1	1
15	15.00	15.00	15.00	15.00	15.00	0.20	0.21	0.21	1	1
16	15.00	15.00	15.00	15.00	15.00	0.20	0.20	0.20	1	1
17	15.00	15.00	15.00	15.00	15.00	0.20	0.20	0.20	1	1
18	15.00	15.00	15.00	15.00	15.00	0.21	0.21	0.21	1	1
19	15.00	15.00	15.00	15.00	15.00	0.21	0.20	0.21	1	1
20	15.00	15.00	15.00	15.00	15.00	0.20	0.20	0.20	1	1
21	15.00	15.00	15.00	15.00	15.00	0.20	0.20	0.03	1	0
22	15.00	15.00	15.00	15.00	15.00	0.20	0.20	0.20	1	1
23	15.00	15.00	15.00	15.00	15.00	0.20	.	.	1	0
24	15.00	15.00	15.00	15.00	15.00	0.20	0.20	0.20	1	1
25	15.00	15.00	15.00	15.00	15.00	.	.	.	0	0
26	15.00	15.00	15.00	15.00	15.00	0.20	0.20	0.12	1	1
27	15.00	15.00	15.00	15.00	15.00	0.17	0.00	0.00	1	0
28	15.00	15.00	15.00	15.00	15.00	0.20	0.20	0.20	1	1

Legend for Table S5: Data are the GCSF and cell dose delivered for all patients randomised to arm 3.

Table S6: Cellular content of apheresis and post-MACS selection product

	Apheresis sample	Post-MACS selection	
			% Recovery
**Total white cell count**	43100.00 (33440.00 - 54060.00)	109.75 (72.60 - 141.13)	0.23 (0.17 - 0.36)
**Total CD34 cell count**	182.35 (109.49 - 225.88)	76.32 (56.35 - 96.54)	44.04 (33.20 - 61.17)
**CD34^{+} cell count/kg**	1.81 (1.30 - 2.41)	0.85 (0.58 - 1.08)	44.04 (33.20 - 61.17)
**Total CD133^{+} cell count**	144.75 (101.28 - 261.98)	74.94 (56.15 - 95.18)	51.33 (34.92 - 70.78)
**CD133^{+} cell count/kg**	1.52 (1.19 - 2.49)	0.85 (0.56 - 1.04)	51.18 (34.92 - 70.78)
**Total CD133^{+} CD45^{low} count**	114.00 (83.88 - 181.46)	74.85 (55.91 - 111.16)	73.32 (51.76 - 84.31)
**CD133^{+} CD45^{low} count/kg**	1.17 (0.92 - 1.72)	0.84 (0.58 - 1.07)	73.32 (51.76 - 84.31)

Legend for Table S6: Cell counts in the apheresis product and after the MACS selection are expressed as medians (IQR). The percentage recovery of cells is detailed in the final column.

Figure S1: Waterfall plot of day 90 change in MELD

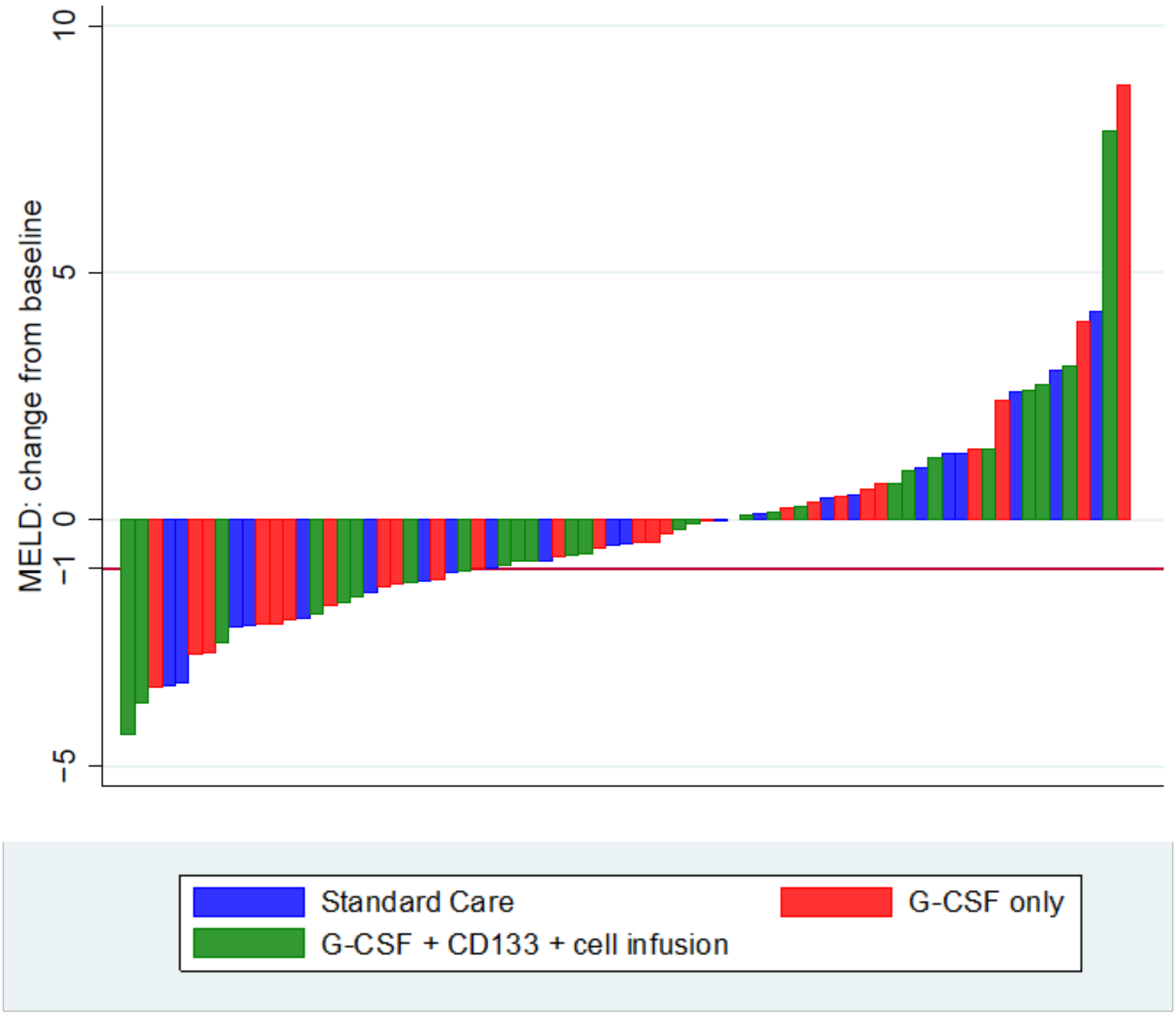

Legend for Figure S1: Waterfall plot of day 90 change in MELD. Each patient's change at day 90 is represented by a bar which has been colour-coded by treatment group. Data were ordered by reduction. Negative values indicate an improvement in MELD score.

Figure S2: Waterfall plot of best change in MELD (to day 90)

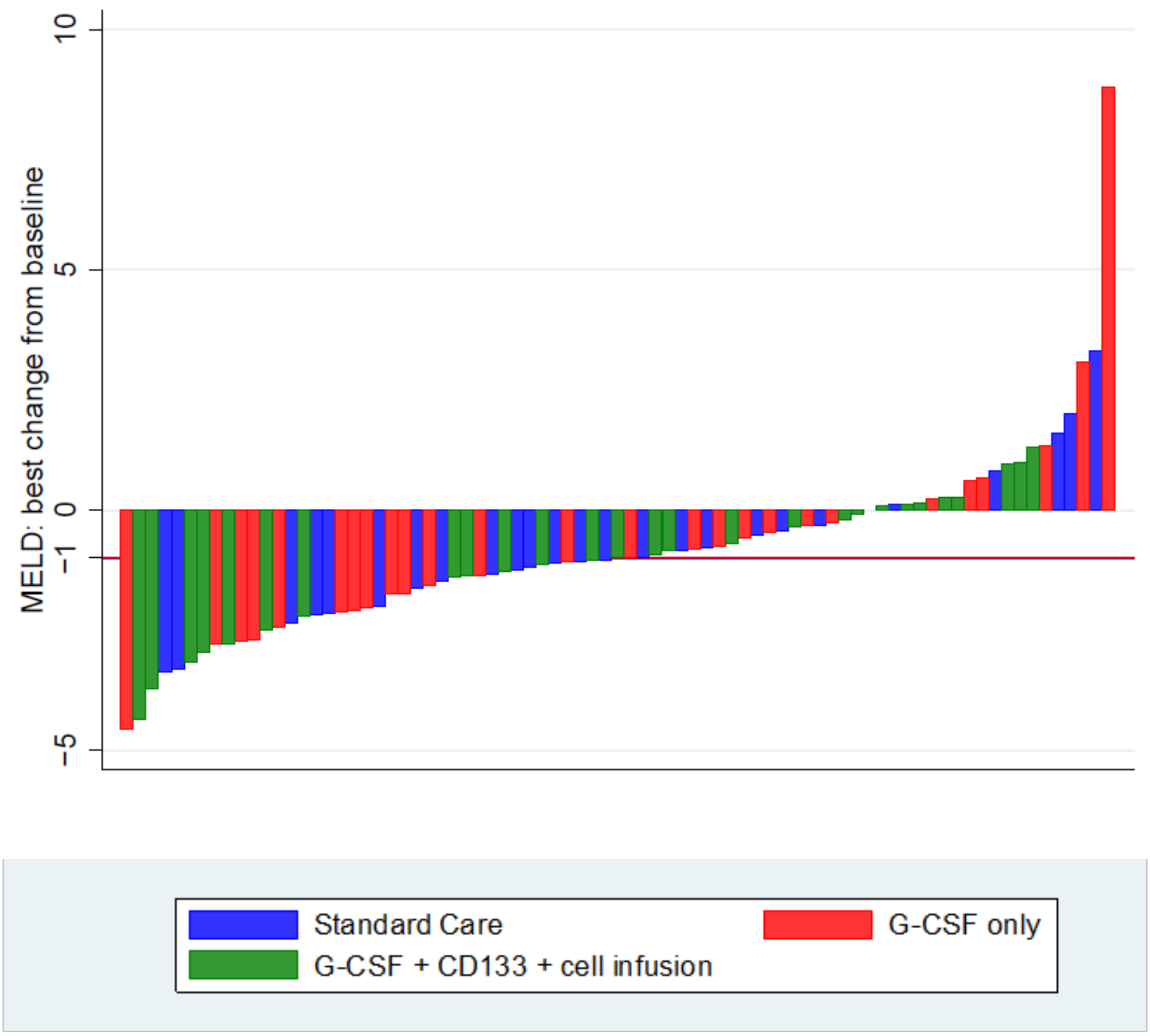

Figure S2: Waterfall plot of best change in MELD (to day 90). Each patient's best change is calculated as the largest reduction overserved over 90 days, and is represented by a bar which has been colour-coded by treatment group. Data were ordered by best reduction. Negative values indicate an improvement in MELD score.

Table S7: Change in MELD and UKELD from baseline to D180 and D360

	Arm 1		Arm 2		Arm 3	
	**Day 180**	**Day 360**	**Day 180**	**Day 360**	**Day 180**	**Day 360**
**Liver disease severity**						
MELD	-0.6 (-1.6, 0.9)	-0.4 (-1.8, 1.0)	0.5 (-0.8, 2.0)	0.2 (-1.6, 1.2)	0.1 (-0.8, 0.6)	0.3 (-0.9, 2.9)
UKELD	0.0 (-2.2, 1.5)	-1.3 (-2.6, 0.9)	-0.2 (-1.0, 1.1)	0.3 (-1.7, 1.8)	0.0 (-1.1, -2.5)	-0.7 (-1.1, 1.4)

Legend for Table S7: Change in MELD and UKELD from baseline to D180 and D360

The table summarises changes in baseline characteristics from day zero to day 180 and day 360. Values presented are delta of medians (IQR). Statistical comparison was made between change at day 180 and 360 to baseline between treatment and control arms – there were no significant differences.

Table **S8: Adverse events**

	Standard Care (28)				G-CSF only (27)				GCSF + cells (26)			
	1/2	3	4	5	1/2	3	4	5	1/2	3	4	5
Investigations	16 (57.1%)	10 (35.7%)	0 (0%)	0 (0%)	16 (59.3%)	10 (37.0%)	1 (3.7%)	0 (0%)	14 (53.8%)	12 (46.2%)	0 (0%)	0 (0%)
Metabolism and nutrition disorders	19 (67.9%)	1 (3.6%)	0 (0%)	0 (0%)	21 (77.8%)	2 (7.4%)	1 (3.7%)	0 (0%)	22 (84.6%)	0 (0%)	0 (0%)	0 (0%)
General disorders and administration site conditions	20 (71.4%)	0 (0%)	0 (0%)	0 (0%)	18 (66.7%)	1 (3.7%)	0 (0%)	0 (0%)	22 (84.6%)	0 (0%)	0 (0%)	0 (0%)
Gastrointestinal disorders	15 (53.6%)	1 (3.6%)	0 (0%)	0 (0%)	19 (70.4%)	2 (7.4%)	1 (3.7%)	0 (0%)	19 (73.1%)	3 (11.5%)	0 (0%)	0 (0%)
Musculoskeletal and connective tissue disorders	13 (46.4%)	1 (3.6%)	0 (0%)	0 (0%)	17 (63.0%)	2 (7.4%)	0 (0%)	0 (0%)	18 (69.2%)	1 (3.8%)	0 (0%)	0 (0%)
Nervous system disorders	8 (28.6%)	1 (3.6%)	1 (3.6%)	0 (0%)	19 (70.4%)	1 (3.7%)	1 (3.7%)	0 (0%)	17 (65.4%)	1 (3.8%)	1 (3.8%)	0 (0%)
Blood and lymphatic system disorders	12 (42.9%)	0 (0%)	0 (0%)	0 (0%)	16 (59.3%)	0 (0%)	0 (0%)	0 (0%)	15 (57.7%)	0 (0%)	0 (0%)	0 (0%)
Respiratory, thoracic and mediastinal disorders	11 (39.3%)	0 (0%)	0 (0%)	0 (0%)	10 (37.0%)	0 (0%)	0 (0%)	0 (0%)	5 (19.2%)	0 (0%)	0 (0%)	0 (0%)
Skin and subcutaneous tissue disorders	7 (25.0%)	0 (0%)	0 (0%)	0 (0%)	6 (22.2%)	0 (0%)	0 (0%)	0 (0%)	5 (19.2%)	1 (3.8%)	0 (0%)	0 (0%)
Psychiatric disorders	4 (14.3%)	1 (3.6%)	0 (0%)	0 (0%)	8 (29.6%)	0 (0%)	0 (0%)	0 (0%)	5 (19.2%)	0 (0%)	0 (0%)	0 (0%)
Infections and infestations	5 (17.9%)	2 (7.1%)	0 (0%)	0 (0%)	4 (14.8%)	0 (0%)	0 (0%)	0 (0%)	4 (15.4%)	3 (11.5%)	0 (0%)	0 (0%)
Hepatobiliary disorders	2 (7.1%)	1 (3.6%)	0 (0%)	0 (0%)	1 (3.7%)	1 (3.7%)	0 (0%)	0 (0%)	6 (23.1%)	0 (0%)	0 (0%)	0 (0%)
Cardiac disorders	-	0 (0%)	0 (0%)	0 (0%)	-	0 (0%)	0 (0%)	0 (0%)	-	1 (3.8%)	0 (0%)	1 (3.8%)
Renal and urinary disorders	-	0 (0%)	0 (0%)	0 (0%)	-	1 (3.7%)	0 (0%)	0 (0%)	-	1 (3.8%)	0 (0%)	0 (0%)
Vascular disorders	-	0 (0%)	0 (0%)	0 (0%)	-	0 (0%)	0 (0%)	0 (0%)	-	1 (3.8%)	0 (0%)	0 (0%)
Surgical and medical procedures	-	2 (7.1%)	0 (0%)	0 (0%)	-	1 (3.7%)	0 (0%)	0 (0%)	-	0 (0%)	0 (0%)	0 (0%)

Legend for Table S8: This table details worst-grade adverse events at the patient level and by CTC category. For grades 1 and 2, the CTC categories reported are those in which 10% or more of patients experienced grade of 1 or 2 at worst. All CTC categories with grades of 3 and above are reported.. The Gastrointestinal adverse events included symptoms such as abdominal pain, ascites, bloating, constipation, diarrhoea, dyspepsia, nausea and vomiting. Musculoskeletal and connective tissue disorders consisted of arthralgia, back pain, generalised muscle weakness, myalgia and pain in extremity. Nervous system disorders consisted of amnesia, dizziness, encephalopathy, headache, lethargy, peripheral neuropathy, syncope and tremor.
